# Timing of cognitive decline onset and life-course trajectories A pooled longitudinal analysis of 14,389 adults across three cohorts

**DOI:** 10.1016/j.tjpad.2026.100649

**Published:** 2026-08-07

**Authors:** Jan Sebastian Novotný, Sivan Klil-Drori, Yonas Endale Geda, Ziad Nasreddine, Gorazd Bernard Stokin

**Affiliations:** a Institute of Molecular and Translational Medicine, Faculty of Medicine and Dentistry, Palacky University Olomouc, Olomouc, Czech Republic; b Department of Psychology and Abnormal Psychology, Faculty of Education, Palacky University Olomouc, Olomouc, Czech Republic; c Department of Psychiatry, McGill University, Montreal, Canada; d MoCA Clinic and Institute, Quebec, Canada; e Department of Neurology, and the Franke Global Neuroscience Education Center, Barrow Neurological Institute, Phoenix AZ, USA; f Department of Neurology, Gloucestershire Hospitals NHS Foundation Trust, Gloucester, UK

**Keywords:** Cognitive ageing, healthy longevity, longitudinal study, midlife, cognition, screening, lifespan trajectories

## Abstract

**Background:**

Population ageing is driving a global surge in cognitive impairment. While late-life decline is well-characterized, the onset of measurable cognitive changes across the full adult lifespan remains uncertain. We aimed to estimate the age at which detectable decline begins using longitudinal data from three population-based cohorts from three countries spanning three continents.

**Methods:**

We analyzed participant-level longitudinal data from the China Health and Retirement Longitudinal Study (CHARLS; China), Midlife in the United States (MIDUS; USA), and Kardiovize (KV; Czech Republic). Cognitive performance was assessed using validated instruments (TICS, BTACT, and MoCA). Scores were harmonized using the percent of maximum possible method (0-100 metric). Linear mixed-effects models, adjusted for age group, sex, and education estimated change over an average 7–9-year follow-up.

**Findings:**

In pooled sample of 14,389 participants (baseline age 22-94 years), a detectable decline in total cognition first emerged in the 31-40 age group (mean change -1.2 points, 95% CI -2.1 to -0.3). Decline magnitude increased progressively with age, reaching -11.4-points in the oldest strata (all p<0.001). Non-memory domains showed earlier vulnerability (detectable from age 31), while significant memory decline emerged after age 50. Individual trajectories showed substantial heterogeneity, suggesting that chronological age is not a deterministic proxy for decline.

**Interpretation:**

Population-level cognitive decline became detectable in early midlife, decades before the traditional clinical focus on older age. Across three population-based cohorts from Asia, North America, and Europe, we observed consistent early decline in non-memory domains followed by later memory decline. These findings support evaluation of midlife cognitive monitoring and life-course approaches to brain health.

**Funding:**

European Union Next Generation EU; European Regional Development Fund; European Social Fund; Barrow Neurological Foundation; NIH.

## Highlights

• Measurable decline in global cognition emerges as early as the fourth decade of life (aged 31-40).
• Cognitive decline shows domain-specific asynchrony: non-memory domains exhibit significant attrition nearly two decades before detectable changes in episodic memory.
• Early monitoring of multidomain cognitive health – rather than a late-life focus on memory alone – may be essential for proactive dementia prevention.

## 1 Introduction

The global focus on dementia prevention has increasingly shifted towards a life-course model, recognizing that modifiable risk factors operating throughout adulthood substantially influence cognitive reserve and the risk of dementia later in life [1,2]. Despite this conceptual shift, however, one fundamental question remains incompletely answered: at what age does measurable cognitive decline first emerge in the general population?

Normal cognitive ageing [3] is a heterogeneous process rather than a uniform decline. Different cognitive domains follow distinct trajectories across adulthood, reflecting differential vulnerability of the underlying neural systems. Longitudinal studies consistently demonstrate that processing speed, executive function, reasoning, and attention tend to decline earlier than episodic memory, although substantial inter-individual variability has been consistently observed [[4], [5], [6], [7], [8], [9], [10], [11], [12]]. Educational attainment, cognitive reserve, vascular and metabolic health, psychiatric disorders, lifestyle factors, and practice effects associated with repeated cognitive testing further influence these trajectories [8,[12], [13], [14], [15], [16], [17], [18]]. Consequently, estimates of both the onset and magnitude of age-related cognitive decline vary considerably across studies.

Although cognitive ageing has been extensively investigated, relatively few longitudinal studies have examined cognitive performance across the full adult lifespan. Most have recruited participants aged 50 years or older, thereby characterising relatively advanced stages of cognitive ageing rather than the earliest stages of cognitive ageing [5,7,13,19,20]. Other studies have focused on specific cognitive domains, intelligence, or selected populations instead of evaluating overall cognitive performance throughout adulthood [5,6,11,19,21,22]. As a result, despite an extensive literature on cognitive ageing, the age at which measurable cognitive decline first becomes detectable remains uncertain [[23], [24], [25]].

An additional limitation of the existing literature is that most studies have been conducted within a single country using a single cognitive assessment instrument. Differences in study populations, educational attainment, language, cultural background, and neuropsychological measures hinder direct comparisons and likely contribute to the substantial variability in reported cognitive trajectories [22,[26], [27], [28], [29], [30], [31]]. Consequently, few studies have compared longitudinal patterns of cognitive ageing across geographically and culturally diverse populations using harmonized analytical methods.

Importantly, the biological processes underlying age-related cognitive disorders begin decades before the clinical onset of mild cognitive impairment (MCI) or dementia [32]. Neuropathological, neuroimaging, and biomarker studies consistently demonstrate that neurodegenerative and cerebrovascular changes accumulate long before overt cognitive impairment becomes clinically apparent [[33], [34], [35], [36], [37], [38]]. If measurable cognitive decline likewise begins substantially earlier than is currently recognized, identifying its onset could have important implications for dementia prevention by informing the optimal timing of interventions aimed at preserving cognitive function and delaying subsequent cognitive impairment.

To address these knowledge gaps, we performed a pooled participant-level longitudinal analysis of harmonised data from three large population-based cohorts representing Asia (CHARLS), North America (MIDUS), and Europe (Kardiovize), comprising 14,389 adults aged 22–94 years. We sought to determine the age at which measurable cognitive decline first becomes detectable at the population level, compare trajectories across memory and non-memory cognitive domains, and evaluate the consistency of these findings across geographically and culturally diverse populations.

## 2 Methods

### 2.1 Study Design and Participants

We conducted a pooled longitudinal analysis using participant-level data from three geographically and culturally diverse population-based cohorts: the China Health and Retirement Longitudinal Study (CHARLS) [39,40], Midlife in the United States (MIDUS) [41], and the Kardiovize (KV) study in the Czech Republic [42]. Participants were eligible if they completed cognitive assessment at both baseline and follow-up.

For all three cohorts, participants were excluded if they had incomplete longitudinal cognitive data or a documented history at baseline of dementia, memory problems, intellectual disability, major neurological disorders likely to affect cognition (e.g., Parkinson’s disease or stroke), or severe psychiatric illness. Dementia and the other exclusion diagnoses were not determined using the cognitive instruments analysed in the present study but were identified from the baseline health information collected within each cohort (self-reported physician diagnosis and/or medical history according to the original cohort protocols). Because harmonized diagnostic criteria for mild cognitive impairment (MCI) were not available across all three cohorts, MCI was not evaluated as a separate diagnostic category. Participants reporting memory problems at baseline were therefore excluded to minimize the inclusion of individuals with possible prodromal cognitive impairment.

All three cohorts systematically collected detailed health information, including medical history, chronic diseases, neurological and psychiatric disorders, medication use, lifestyle factors, and functional status. In the present study, these data were used solely to apply the predefined exclusion criteria and were not included as covariates in the analyses.

The CHARLS dataset is a nationally representative longitudinal survey initiated in 2011 that collected information on sociodemographic characteristics, socioeconomic status, and health [39,40]. A stratified multistage probability-proportional-to-size sampling design was used to recruit participants from 450 villages or urban communities across 150 counties or districts in 28 Chinese provinces. We analyzed data from Wave 1 (2011) and Wave 4 (2018). After excluding 437 participants who met the predefined exclusion criteria, 11,639 participants with complete longitudinal cognitive data were included (mean age 57.4±8.7 years; 6,354 [54.6%] women).

The MIDUS dataset is a national longitudinal study of health and well-being based on a probability sample designed to be broadly representative of the non-institutionalized adult population of the United States [41]. We analyzed data from MIDUS II (2004-2006) and MIDUS III (2013). After excluding 503 participants who met the predefined exclusion criteria, the final sample comprised 2,442 participants (mean age 54.3±10.9 years; 1,363 [55.8%] women).

The KV study is a longitudinal epidemiological cohort based on a randomly selected representative 1% sample of residents of Brno, Czech Republic, established to investigate major health-related issues in Central Europe [42]. Baseline assessments were conducted between 2013 and 2015, with follow-up between 2021 and 2022. After excluding 12 participants who met the predefined exclusion criteria, 296 participants with complete longitudinal data were included (mean age 47.1±10.2; 139 [47%] women).

Overall, the pooled dataset comprised 14,389 adults aged 22 to 94 years with a mean follow-up duration of 8^.^1 years (range 7-9 years).

### 2.2 Cognitive Assessment and Harmonization

Global cognitive function was evaluated using three validated instruments: the Telephone Interview of Cognitive Status (TICS-10) [43,44] in CHARLS, the Brief Test of Adult Cognition by Telephone (BTACT) [45] in MIDUS, and Montreal Cognitive Assessment (MoCA) [46,47] in KV. The cognitive domains assessed differed somewhat across the three instruments. TICS-10 evaluates orientation, memory, attention/executive function, and visuospatial ability; BTACT assesses memory, attention/executive function, and visuospatial ability; and MoCA evaluates memory, attention/executive function, language, visuospatial ability, and orientation. Overall, the overlap of the cognitive domains assessed by the three instruments was sufficient to permit harmonization (Supplementary Figure 1). At the item level, the memory domain was comparable across instruments, with all three including similar episodic memory tasks. In contrast, the non-memory domains differed in both the number and emphasis of individual items. Therefore, rather than attempting direct item-by-item comparisons, we derived a composite non-memory cognitive score within each instrument by averaging the relevant items before harmonization.

To address the heterogeneity in administration mode and scoring ranges, cognitive scores were harmonized using the Percent of Maximum Possible (POMP) method [48,49]. POMP scores transform raw values into a 0-100 metric using the formula: *POMP* = ((observed – minimum) / (maximum – minimum)) x 100. This approach preserves the original distribution and variance of each instrument while enabling cross-cohort aggregation. Primary outcomes were defined as harmonized total cognition, and two construct-level sub-domains: memory (e.g., word recall) and non-memory (e.g., attention, orientation, language, and executive function).

Most cognitive assessment instruments do not define criteria for the onset of normal cognitive decline but instead provide thresholds for clinically significant cognitive impairment. Furthermore, there is currently no consensus regarding what constitutes meaningful cognitive decline during normal ageing (e.g., a predefined decrease in test score, a change exceeding a specified standard deviation, a score below an age-adjusted normative threshold, or deterioration in daily functioning). Therefore, we defined the onset of cognitive decline as the earliest statistically significant reduction in the harmonized cognitive score between baseline and follow-up (approximately 8 years). This longitudinal, population-based definition is consistent with previous studies and identifies the earliest age at which cognitive decline becomes detectable at the population level rather than clinically meaningful impairment in an individual.

### 2.3 Statistical analysis

We employed linear mixed-effects regression models with participant-specific random intercepts to account for the nested structure of longitudinal data and inter-individual variability at baseline. The primary independent variables were follow-up interval (years) and baseline age group (categorized in 10-year increments). A pooled participant-level dataset was created by combining the harmonized data from the three cohorts (CHARLS, MIDUS, and KV). No weighting was applied because the analyses were performed at the individual participant level, with each participant contributing equally to the pooled estimates; consequently, the contribution of each cohort reflected its sample size. To evaluate whether the larger size of the CHARLS cohort disproportionately influenced the pooled results, we performed sensitivity analyses by repeating all primary analyses separately within each cohort and comparing the resulting age-stratified trajectories with those obtained from the pooled dataset.

Models were adjusted for sex and educational attainment (dichotomized as ≤12 vs ≥12 years). To estimate the onset of decline, we included interaction terms between age groups and follow-up interval. These interaction coefficients represent annualized rate of change specific to each decade of life. Statistical significance was set at α=0.05 (two-tailed). As most participants provided two data points, models estimated average interval change rather than non-linear individual trajectories. All analyses were conducted in R (v4.2.1) using RStudio (v2022.07.2).

## 3 Results

### 3.1 Cohort Characteristics

The pooled sample (n=14 389) provided broad demographic representation across China, the USA, and Central Europe. Baseline ages spanned seven decades (22-94 years). Educational attainment was diverse, and mean follow-up duration was 8.1 years (SD 1.4) (Table 1).

**Table 1:** Baseline characteristics and study design parameters of included longitudinal cohorts.

	**Study population**					**Cognitive measure**	
**Study**	**N**	**Study range (used)**	**Age range (baseline)**	**Location**	**Representativeness**	**Name**	**Range**
CHARLS	11639	2011-2018	22-92 years	China	Population-based study	TICS	0-31
MIDUS	2442	2004-2013	33-83 years	USA	Population-based study	BTACT	0-160
KV	296	2013-2022	33-65 years	Czech Republic	Population-based study	MoCA	0-30

### 3.2 The Onset of Global Decline

**Figure 1:**
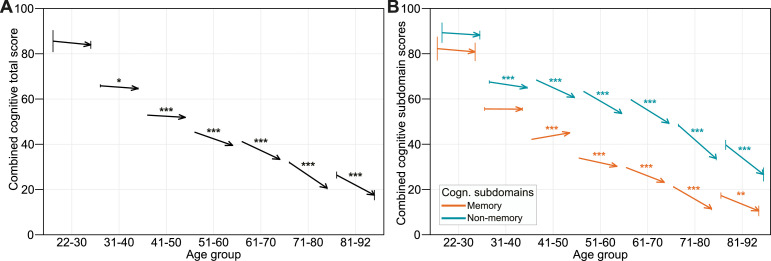
Longitudinal cognitive trajectories and domain-specific changes across the adult lifespan.

Longitudinal pooled model revealed that cognitive performance does not remain stable until old age. Instead, a statistically significant negative slope was observed beginning in the 31–40-year age group. While the initial drop was modest (mean change -1.2 points, 95% CI -2.1 to -0.3), the rate of decline accelerated sharply after age 60, indicating a non-linear acceleration of cognitive attrition over the lifespan (Figure 1A, Supplementary Table S2).

(**A**) Estimated changes in harmonized total cognitive performance (0-100) by baseline age group. (**B**) Estimated longitudinal changes in memory and non-memory domains by baseline age group. Values represent estimated mean changes over a mean follow-up of approximately 8 years, derived from linear mixed-effects model adjusted for sex and education. Arrows indicate the direction and magnitude of change from baseline to follow-up, and vertical bars denote standard errors of the mean (SEM). Asterisks indicate statistically significant changes (P ≤ 0.05, P ≤ 0.01, P ≤ 0.001).

### 3.3 Domain Asynchrony

The pooled data revealed a clear temporal gap between domain-specific declines (Figure 1B). Performance in non-memory domains, including attention, orientation, language, and executive function began to drop in the early 30s. Significant decline in memory was largely absent in younger cohorts, only becoming statistically evident after age 50.

### 3.4 Heterogeneity and Sensitivity

**Figure 2:**
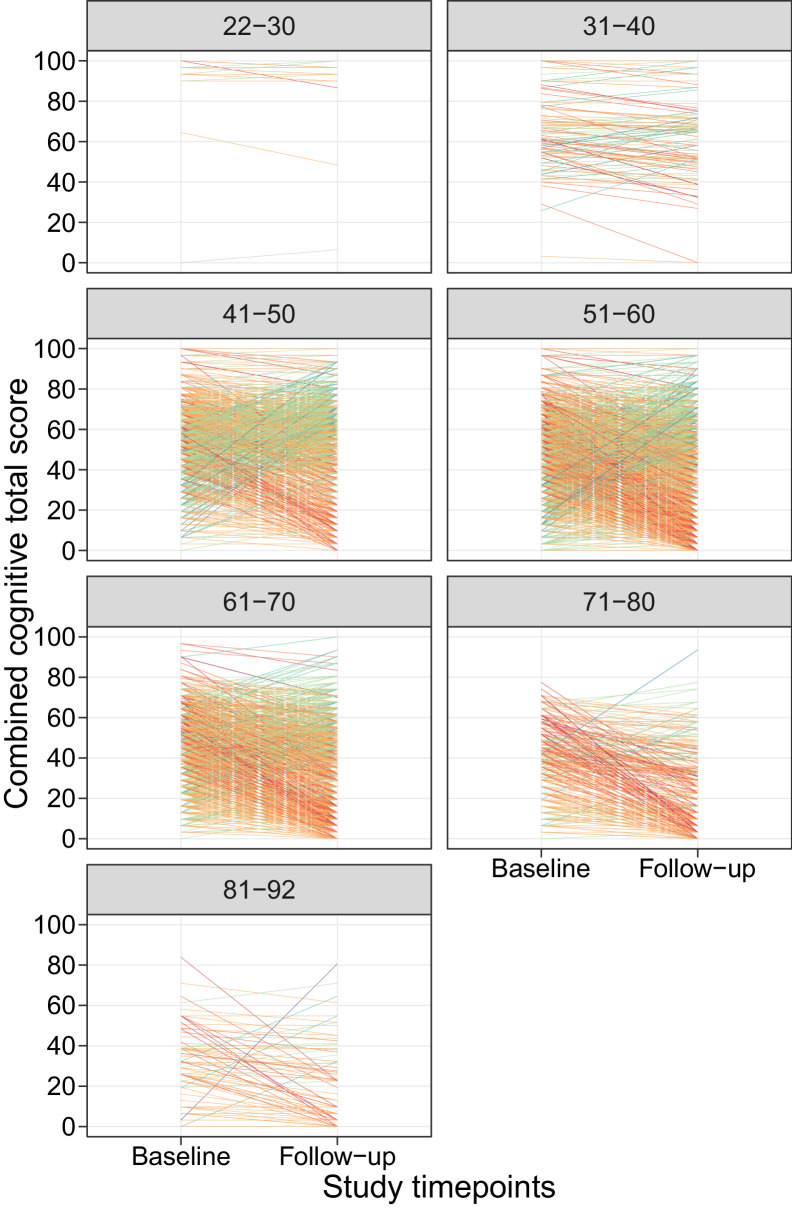
Heterogeneity of individual cognitive trajectories during follow-up.

Individual-level “spaghetti plots” showed that, while the population mean exhibited a downward trend, individual trajectories remained highly heterogeneous (Figure 2). Sensitivity analyses performed separately within each cohort demonstrated a consistent pattern of early decline in the non-memory cognitive domain (with changes emerging as early as around 30 years of age) and later decline in memory across all three cohorts (CHARLS, MIDUS, and KV). In contrast, the onset of decline in total cognitive performance occurred later in the individual cohort analyses than in the pooled analysis, with the exception of the U.S. cohort. These findings indicate that, despite some between-cohort differences in the timing of changes in total cognition, the overall pattern of cognitive ageing was consistent across cohorts regardless of the cognitive instrument used or the language of administration (Supplementary Figures 2–4; Supplementary Tables S3–S5).

Spaghetti plot showing participant-level trajectories of harmonized total cognitive scores from baseline to follow-up. Despite an overall population-level decline in cognitive performance, substantial inter-individual variability is evident, with some participants showing stable or improved cognitive performance over time.

## 4 Discussion

To our knowledge, this is among the first participant-level pooled longitudinal analyses to examine cognitive ageing across the adult lifespan using harmonized data from geographically and culturally diverse population-based cohorts. Across three independent cohorts representing Asia, North America, and Europe, we observed a remarkably consistent pattern in which measurable decline in non-memory cognitive domains preceded detectable decline in memory. Although the precise age at which decline in total cognition became statistically detectable varied somewhat between cohorts, the overall temporal sequence remained consistent despite differences in populations, languages, and cognitive instruments. These findings suggest that the earliest detectable population-level cognitive changes may occur substantially earlier in adulthood than traditionally appreciated.

### 4.1 The Earliest Detectable Cognitive Change

The modest decline (1.2-points) observed in the pooled analysis among individuals aged 31-40 years represents the earliest, subclinical stage of cognitive ageing. Although the precise onset of decline in total cognitive performance differed somewhat across individual cohorts, the overall patterns of early non-memory decline followed by later memory decline was consistent. These findings suggest that measurable cognitive changes may begin earlier in adulthood than traditionally assumed and are consistent with previous longitudinal studies reporting early age-related cognitive decline [8,10,[23], [24], [25],^27^,^32^,^50^]. While such a minor change in magnitude is unlikely to result in clinically meaningful functional impairment, it represents a detectable departure from peak cognitive performance at the population level. Importantly, these findings should not be interpreted as indicating clinically meaningful impairment in individuals in their thirties, but rather as identifying the earliest age group in which a small average decline became detectable at the population level. Notably, because repeated cognitive testing generally produces practice effects that tend to improve over time [13], the observed negative longitudinal slope in younger adults is likely to represent a conservative estimate of biological cognitive ageing.

### 4.2 Domain Asynchrony and Heterogeneity

The earlier decline of non-memory compared to memory domains was consistent with previous work indicating that the first cognitive changes in adulthood occur in areas such as attention, executive functions, visuospatial abilities and processing speed, while memory remains intact until later [9,10,13,50]. Our observation that non-memory domains decline before memory is therefore consistent with the hypothesis that age-related alterations in distributed frontostriatal and white matter networks precede substantial involvement of hippocampal memory systems. These findings suggest that the determinants of cognitive changes, particularly during midlife, are likely more heterogeneous than those operating later in life and may include conditions such as anxiety [51], depression [[52], [53], [54]], psychiatric illnesses [55,56], functional cognitive disorders [57,58], cerebral vascular diseases [18,[59], [60], [61]], traumatic brain injury [62,63], as well as systemic causes such as obesity [64] and diabetes [54,65,66].

These observations may be interpreted within the framework of the scaffolding theory of aging and cognition, which proposes that brain networks supporting executive function, including white matter and frontostriatal circuits, may be particularly susceptible to the effects of vascular and metabolic ageing [67,68]. Likewise, the long-standing frontal lobe hypothesis of cognitive aging posits that attention, executive functions, and related cognitive processes depend on prefrontal and frontostriatal networks, which are disproportionately affected by age-related changes [69]. This concept is supported by longitudinal neuroimaging studies demonstrating earlier deterioration of frontal network integrity than hippocampal atrophy [70]. Together, these findings suggest that the earlier decline in non-memory cognition may reflect the greater vulnerability of white matter changes and frontal networks to age-related vascular and metabolic processes. If confirmed, our findings further imply that multidomain cognitive screening may be more informative during midlife than approaches focused primarily on memory assessment.

An equally important finding was the marked heterogeneity of individual cognitive trajectories. Although average cognitive performance declined with age, many participants maintained stable cognition and a subset even improved over time. These observations indicate that chronological age alone is a poor predictor of cognitive trajectory and support the concepts of cognitive reserve, resilience, and differential susceptibility to environmental, vascular, metabolic, and lifestyle factors [32,71,72]. They also demonstrate that cognitive ageing is not a uniform or inevitably progressive process, underscoring the value of longitudinal monitoring over cross-sectional assessment. Identifying the biological and environmental determinants of preserved or improved cognitive performance may prove as informative as studying cognitive decline itself and could reveal novel targets for interventions aimed at maintaining cognitive health throughout adulthood [30].

### 4.3 Public Health and Clinical Practice

Current models of cognitive care remain largely reactive, with assessment typically initiated only after symptomatic impairment has emerged. Our findings support consideration of a life-course approach to brain health in which cognitive monitoring is incorporated into routine midlife preventive care alongside established cardiovascular and metabolic risk assessment, such as blood pressure, glucose, and cholesterol screening [73]. This concept is consistent with the World Health Organization's guidelines on risk reduction of cognitive decline and dementia, many of which are already actionable in midlife [74]. Furthermore, multidomain lifestyle interventions targeting several of these risk factors simultaneously have demonstrated efficacy in randomized controlled trials among older adults at increased risk of dementia, and there is growing recognition that initiating such interventions earlier in adulthood may yield even greater benefits [75].

Although further research is needed to define optimal screening intervals, and implementation strategies, our findings suggest that identifying individuals with accelerated cognitive decline during midlife may enable timely, personalized interventions before clinically significant neurodegeneration becomes established. This is particularly relevant given that up to 45% of dementia cases have been attributed to potentially modifiable risk factors [75]. Translating these findings into practice will require collaboration among researchers, clinicians, policymakers, and international organizations. By demonstrating that measurable cognitive decline is detectable at the population level from the fourth decade of life, our study provides empirical support for evaluating whether brief, validated multidomain cognitive screening should become part of routine midlife preventive healthcare and future life-course dementia prevention strategies [75,76].

## 5 Limitations

Our study has several limitations. First, the three cohorts used different cognitive instruments (TICS, BTACT, MoCA), introducing potential measurement heterogeneity. Although we harmonized cognitive scores using the POMP method to enable cross-cohort comparisons, residual differences in instrument sensitivity, domain composition, and floor to ceiling effects cannot be completely excluded. Alternative harmonization approaches, including latent variable models, were not feasible because of the limited overlap of cognitive items, differences in domain structure, and the small number of items contributing to some domains. Similarly, missing-value imputation was inappropriate because of the absence of overlapping items and the need to preserve longitudinal consistency, whereas z-standardization and ipsatization are generally unsuitable for longitudinal analyses. Importantly, the consistent age-related patterns observed across three independent cohorts suggest that the findings reflect biological trend rather than instrument-specific noise. Second, most participants contributed only two cognitive assessments over a 7-9-year follow-up, limiting our ability to model nonlinear within-person trajectories or distinguish short-term fluctuations from sustained cognitive change. In addition, repeated cognitive testing is susceptible to practice effects. Because repeated testing generally improves performance, the decline observed in younger adults is likely to represent a conservative estimate of the underlying biological change. Third, although analyses were adjusted for age, sex, and education, other potentially important confounders – including cerebrovascular risk factors, mental health, and socioeconomic changes – could not be harmonized across all cohorts and were therefore not included in the primary pooled analyses. Fourth, attrition is an inherent limitation of longitudinal studies. If participants experiencing more rapid cognitive decline were more likely to withdraw, survivor bias may have resulted in underestimation of cognitive decline, particularly at older ages. Finally, although statistically significant at the population level, the modest changes observed in early midlife should not be interpreted as clinically meaningful impairment in individual participants. Rather, they identify a population-level inflection point that warrants further investigation, not a diagnostic threshold for disease.

## 6 Conclusions

Measurable cognitive decline is not an exclusive hallmark of later life but becomes detectable at the population level in early midlife. The early vulnerability of non-memory domains represents a shift in our understanding of cognitive ageing, suggesting that executive and attentional functions may be measurably affected before episodic memory at the population level. These findings provide empirical support for a life-course approach to brain health. Rather than focusing screening and intervention efforts primarily on older adults, healthcare systems should consider midlife as an important window for risk-factor modification and cognitive monitoring. Earlier identification of accelerated cognitive decline may help extend the period of optimal cognitive function and delay the onset of clinically significant impairment through timely preventive interventions. Realizing this life-course approach will require coordinated action by researchers, clinicians, policymakers, and public health organizations. We encourage international organizations, including Alzheimer's Disease International and the World Health Organization, to consider whether future dementia risk-reduction strategies and public health guidance should place greater emphasis on midlife populations, where preventive interventions may have the greatest long-term impact.

## Statements

### Ethics approval

All procedures complied with the ethical standards of the relevant national and institutional committees and with the Declaration of Helsinki. All participants provided written informed consent. Relevant institutional approvals were obtained within each participating cohort: CHARLS (Peking University), MIDUS (University of Wisconsin-Madison), and KV (St. Anne’s University Hospital, Brno).

### Role of the funding source

The funders of the study had no role in study design, data collection, data analysis, interpretation, writing of the report, or the decision to submit the paper for publication. The corresponding author had full access to all the data in the study and had final responsibility for the decision to submit for publication.

### Contributors

GBS conceived the study. JSN and GBS designed the analysis. JSN performed the formal statistical analyses and data visualization. JSN and GBS drafted the initial manuscript. SKD, YEG, and ZN critically revised the manuscript for important intellectual content. All authors have full access to the data, interpreted the results, and approved the final version for submission. GBS and JSN have verified the underlying data.

### Data sharing

Access to participant-level data is subject to the data use agreements and governance policies of the original cohort studies: CHARLS (charls.pku.edu.cn), MIDUS (midus.wisc.edu), and KV. Derived analytical code and additional methodological details are available from the corresponding author on reasonable request, subject to institutional approvals. And data transfer agreements

### Reporting standards

This study is reported in accordance with the Strengthening the Reporting of Observational Studies in Epidemiology (STROBE) guidelines.

### Funding

The work was supported by the European Union Next Generation EU programme (LX22NPO5107 [MEYS]), the European Regional Development Fund and the European Social Fund (CZ.02.1.01/0.0/0.0/16_019/0000868), the Barrow Neurological Foundation, and the US National Institutes of Health (R01AG057708).

### Declaration of generative AI and AI-assisted technologies in the manuscript preparation process

During the preparation of this manuscript, the authors used ChatGPT (OpenAI) to assist with language editing, improving clarity, grammar, and manuscript organization. The AI tool was not used to generate, analyze, or interpret scientific data, perform statistical analyses, or draw scientific conclusions. All scientific content, interpretation, and final editorial decisions were made by the authors, who take full responsibility for the accuracy and integrity of the manuscript.

## CRediT authorship contribution statement

**Jan Sebastian Novotný:** Writing – original draft, Visualization, Methodology, Investigation, Formal analysis, Data curation. **Sivan Klil-Drori:** Writing – review & editing. **Yonas Endale Geda:** Writing – review & editing. **Ziad Nasreddine:** Writing – review & editing. **Gorazd Bernard Stokin:** Writing – original draft, Validation, Supervision, Methodology, Funding acquisition, Data curation, Conceptualization.

## Declaration of competing interest

The authors declare the following financial interests/personal relationships which may be considered as potential competing interests:

ZN is the creator of the MoCA and is associated with its development and dissemination. All other authors declare no competing interests.

## Appendix Supplementary materials

Image, application 1

Image, application 2

Image, application 3

Image, application 4

Image, application 5

Image, application 6

## References

[1] Livingston G. (2020). Dementia prevention, intervention, and care: 2020 report of the Lancet Commission. Lancet.

[2] Orgeta V. (2019). The Lancet Commission on Dementia Prevention, Intervention, and Care: a call for action. Ir J Psychol Med.

[3] Atchley R.C. (1989). A continuity theory of normal aging. Gerontologist.

[4] Harada C.N., Natelson Love M.C., Triebel K.L. (2013). Normal cognitive aging. Clin Geriatr Med.

[5] Finkel D. (2007). Age changes in processing speed as a leading indicator of cognitive aging. Psychol Aging.

[6] Tucker-Drob E.M., Johnson K.E., Jones R.N. (2009). The cognitive reserve hypothesis: a longitudinal examination of age-associated declines in reasoning and processing speed. Dev Psychol.

[7] Li G., Li K. (2022). Turning Point of Cognitive Decline for Chinese Older Adults from a Longitudinal Analysis: Protective Factors and Risk Factors. Healthcare (Basel).

[8] Yang Y.C. (2024). An Early and Unequal Decline: Life Course Trajectories of Cognitive Aging in the United States. J Aging Health.

[9] Ronnlund M. (2005). Stability, growth, and decline in adult life span development of declarative memory: cross-sectional and longitudinal data from a population-based study. Psychol Aging.

[10] Salthouse T.A. (2019). Trajectories of normal cognitive aging. Psychol Aging.

[11] Schaie K.W. (1994). The course of adult intellectual development. Am Psychol.

[12] Cabeza R. (2018). Maintenance, reserve and compensation: the cognitive neuroscience of healthy ageing. Nat Rev Neurosci.

[13] Salthouse T.A. (2010). Influence of age on practice effects in longitudinal neurocognitive change. Neuropsychology.

[14] Deary I.J., Penke L., Johnson W. (2010). The neuroscience of human intelligence differences. Nat Rev Neurosci.

[15] Jyrkka J. (2011). Association of polypharmacy with nutritional status, functional ability and cognitive capacity over a three-year period in an elderly population. Pharmacoepidemiol Drug Saf.

[16] McTeague L.M., Goodkind M.S., Etkin A. (2016). Transdiagnostic impairment of cognitive control in mental illness. J Psychiatr Res.

[17] Nyberg L., Pudas S. (2019). Successful Memory Aging. Annu Rev Psychol.

[18] van Nieuwkerk A.C. (2023). Cognitive Impairment in Patients With Cardiac Disease: Implications for Clinical Practice. Stroke.

[19] Folia V. (2022). Longitudinal trajectories and normative language standards in older adults with normal cognitive status. Neuropsychology.

[20] Hughes M.L. (2018). Change in Cognitive Performance From Midlife Into Old Age: Findings from the Midlife in the United States (MIDUS) Study. J Int Neuropsychol Soc.

[21] Park D.C. (2002). Models of visuospatial and verbal memory across the adult life span. Psychol Aging.

[22] Stinchcombe A., Hammond N.G., Hopper S. (2023). Changes in executive function in the Canadian longitudinal study on aging over 3-years: A focus on social determinants of health. Front Psychol.

[23] Salthouse T.A. (2009). When does age-related cognitive decline begin?. Neurobiol Aging.

[24] Dohm-Hansen S. (2024). The 'middle-aging' brain. Trends Neurosci.

[25] Elliott M.L. (2021). Brain-age in midlife is associated with accelerated biological aging and cognitive decline in a longitudinal birth cohort. Mol Psychiatry.

[26] Singh-Manoux A. (2012). Timing of onset of cognitive decline: results from Whitehall II prospective cohort study. BMJ.

[27] Karlamangla A.S. (2017). Evidence for Cognitive Aging in Midlife Women: Study of Women's Health Across the Nation. PLoS One.

[28] Lyketsos C.G., Chen L.S., Anthony J.C. (1999). Cognitive decline in adulthood: an 11.5-year follow-up of the Baltimore Epidemiologic Catchment Area study. Am J Psychiatry.

[29] Schaie K.W., Willis S.L., Caskie G.I. (2004). The Seattle longitudinal study: relationship between personality and cognition. Neuropsychol Dev Cogn B Aging Neuropsychol Cogn.

[30] Zaninotto P. (2018). Cognitive function trajectories and their determinants in older people: 8 years of follow-up in the English Longitudinal Study of Ageing. J Epidemiol Community Health.

[31] Jonaitis E.M. (2019). Measuring longitudinal cognition: Individual tests versus composites. Alzheimers Dement (Amst).

[32] Caselli R.J. (2020). Neuropsychological decline up to 20 years before incident mild cognitive impairment. Alzheimers Dement.

[33] Bateman R.J. (2012). Clinical and biomarker changes in dominantly inherited Alzheimer's disease. N Engl J Med.

[34] Li Y. (2024). Timing of Biomarker Changes in Sporadic Alzheimer's Disease in Estimated Years from Symptom Onset. Ann Neurol.

[35] Bennett I.J., Madden D.J. (2014). Disconnected aging: cerebral white matter integrity and age-related differences in cognition. Neuroscience.

[36] Villemagne V.L. (2013). Amyloid beta deposition, neurodegeneration, and cognitive decline in sporadic Alzheimer's disease: a prospective cohort study. Lancet Neurol.

[37] Sperling R.A. (2011). Toward defining the preclinical stages of Alzheimer's disease: recommendations from the National Institute on Aging-Alzheimer's Association workgroups on diagnostic guidelines for Alzheimer's disease. Alzheimers Dement.

[38] Price J.L., Morris J.C. (1999). Tangles and plaques in nondemented aging and "preclinical" Alzheimer's disease. Ann Neurol.

[39] Zhao Y., Strauss J., Yang G. (2013).

[40] Zhao Y., Strauss J., Chen X. (2020).

[41] Radler B.T. (2014).

[42] Movsisyan N.K. (2018). Kardiovize Brno 2030, a prospective cardiovascular health study in Central Europe: Methods, baseline findings and future directions. Eur J Prev Cardiol.

[43] Crimmins E.M. (2011). Assessment of cognition using surveys and neuropsychological assessment: the Health and Retirement Study and the Aging, Demographics, and Memory Study. J Gerontol B Psychol Sci Soc Sci.

[44] de Jager C.A., Budge M.M., Clarke R. (2003). Utility of TICS-M for the assessment of cognitive function in older adults. Int J Geriatr Psychiatry.

[45] DiBlasio C.A. (2021). Research Letter: Performance of the Brief Test of Adult Cognition by Telephone in a National Sample. J Head Trauma Rehabil.

[46] Nasreddine Z.S. (2005). The Montreal Cognitive Assessment, MoCA: a brief screening tool for mild cognitive impairment. J Am Geriatr Soc.

[47] Julayanont P. (2014). Montreal Cognitive Assessment Memory Index Score (MoCA-MIS) as a predictor of conversion from mild cognitive impairment to Alzheimer's disease. J Am Geriatr Soc.

[48] Cohen P., Cohen J., Aiken LS., West S.G. (1999). The problem of units and the circumstance for POMP. Multivariate Behavioral Research.

[49] Moeller J. (2015). A word on standardization in longitudinal studies: don't. Front Psychol.

[50] Tucker-Drob E.M. (2011). Global and domain-specific changes in cognition throughout adulthood. Dev Psychol.

[51] Gulpers B. (2016). Anxiety as a Predictor for Cognitive Decline and Dementia: A Systematic Review and Meta-Analysis. Am J Geriatr Psychiatry.

[52] Desai R. (2020). Temporal Relationship Between Depressive Symptoms and Cognition in Mid and Late Life: A Longitudinal Cohort Study. J Am Med Dir Assoc.

[53] Chan C.K. (2020). Depressive symptoms and CSF Alzheimer's disease biomarkers in relation to clinical symptom onset of mild cognitive impairment. Alzheimers Dement (Amst).

[54] Chow Y.Y. (2022). Associations between depression and cognition, mild cognitive impairment and dementia in persons with diabetes mellitus: A systematic review and meta-analysis. Diabetes Res Clin Pract.

[55] Trivedi J.K. (2006). Cognitive deficits in psychiatric disorders: Current status. Indian J Psychiatry.

[56] Millan M.J. (2012). Cognitive dysfunction in psychiatric disorders: characteristics, causes and the quest for improved therapy. Nat Rev Drug Discov.

[57] Pennington C. (2015). Functional cognitive disorder: what is it and what to do about it?. Pract Neurol.

[58] Kemp S. (2022). Functional Cognitive Disorder: Differential Diagnosis of Common Clinical Presentations. Arch Clin Neuropsychol.

[59] Knopman D.S. (2018). Midlife vascular risk factors and midlife cognitive status in relation to prevalence of mild cognitive impairment and dementia in later life: The Atherosclerosis Risk in Communities Study. Alzheimers Dement.

[60] Yaneva-Sirakova T. (2017). Screening for mild cognitive impairment in patients with cardiovascular risk factors. Neuropsychiatr Dis Treat.

[61] Arce Renteria M. (2022). Midlife Vascular Factors and Prevalence of Mild Cognitive Impairment in Late-Life in Mexico. J Int Neuropsychol Soc.

[62] LoBue C. (2018). Traumatic brain injury history and progression from mild cognitive impairment to Alzheimer disease. Neuropsychology.

[63] Li W. (2016). Traumatic brain injury and age at onset of cognitive impairment in older adults. J Neurol.

[64] Qu Y. (2020). Association of body mass index with risk of cognitive impairment and dementia: A systematic review and meta-analysis of prospective studies. Neurosci Biobehav Rev.

[65] Cukierman T., Gerstein H.C., Williamson J.D. (2005). Cognitive decline and dementia in diabetes–systematic overview of prospective observational studies. Diabetologia.

[66] Gudala K. (2013). Diabetes mellitus and risk of dementia: A meta-analysis of prospective observational studies. J Diabetes Investig.

[67] Park D.C., Reuter-Lorenz P. (2009). The adaptive brain: aging and neurocognitive scaffolding. Annu Rev Psychol.

[68] Reuter-Lorenz P.A., Park D.C. (2014). How does it STAC up? Revisiting the scaffolding theory of aging and cognition. Neuropsychol Rev.

[69] West R.L. (1996). An application of prefrontal cortex function theory to cognitive aging. Psychol Bull.

[70] Fjell A.M. (2016). Brain Events Underlying Episodic Memory Changes in Aging: A Longitudinal Investigation of Structural and Functional Connectivity. Cereb Cortex.

[71] van der Willik K.D. (2021). Trajectories of Cognitive and Motor Function Between Ages 45 and 90 Years: A Population-Based Study. J Gerontol A Biol Sci Med Sci.

[72] Soldan A. (2017). Cognitive reserve and long-term change in cognition in aging and preclinical Alzheimer's disease. Neurobiol Aging.

[73] Mukadam N. (2024). Benefits of population-level interventions for dementia risk factors: an economic modelling study for England. Lancet Healthy Longev.

[74] Chowdhary N. (2021). Reducing the Risk of Cognitive Decline and Dementia: WHO Recommendations. Front Neurol.

[75] Ngandu T. (2015). A 2 year multidomain intervention of diet, exercise, cognitive training, and vascular risk monitoring versus control to prevent cognitive decline in at-risk elderly people (FINGER): a randomised controlled trial. Lancet.

[76] Long S, B.C., Weidner W, World Alzheimer Report 2023: Reducing Dementia Risk — Never Too Early, Never Too Late. 2023.

