# Supplementary material for: Timing of cognitive decline onset and life-course trajectories A pooled longitudinal analysis of 14,389 adults across three cohorts
Source: J Prev Alzheimers Dis. 2026 Aug 7;13(9):100649. doi: 10.1016/j.tjpad.2026.100649 (PMC13475508; doi:10.1016/j.tjpad.2026.100649)
Supplement: Supplementary file 1 [file mmc3.pdf]

Cognitive domain	Cognitive tool		
	TICS-10	BTACT	MoCA
Memory	●	●	●
Attention/ Executive functions	●	●	●
Language/ Fluency		●	●
Orientation	●		●
Visuospatial abilities	●	●	●
