# Supplementary material for: Timing of cognitive decline onset and life-course trajectories A pooled longitudinal analysis of 14,389 adults across three cohorts
Source: J Prev Alzheimers Dis. 2026 Aug 7;13(9):100649. doi: 10.1016/j.tjpad.2026.100649 (PMC13475508; doi:10.1016/j.tjpad.2026.100649)
Supplement: Supplementary file 2 [file mmc4.pdf]

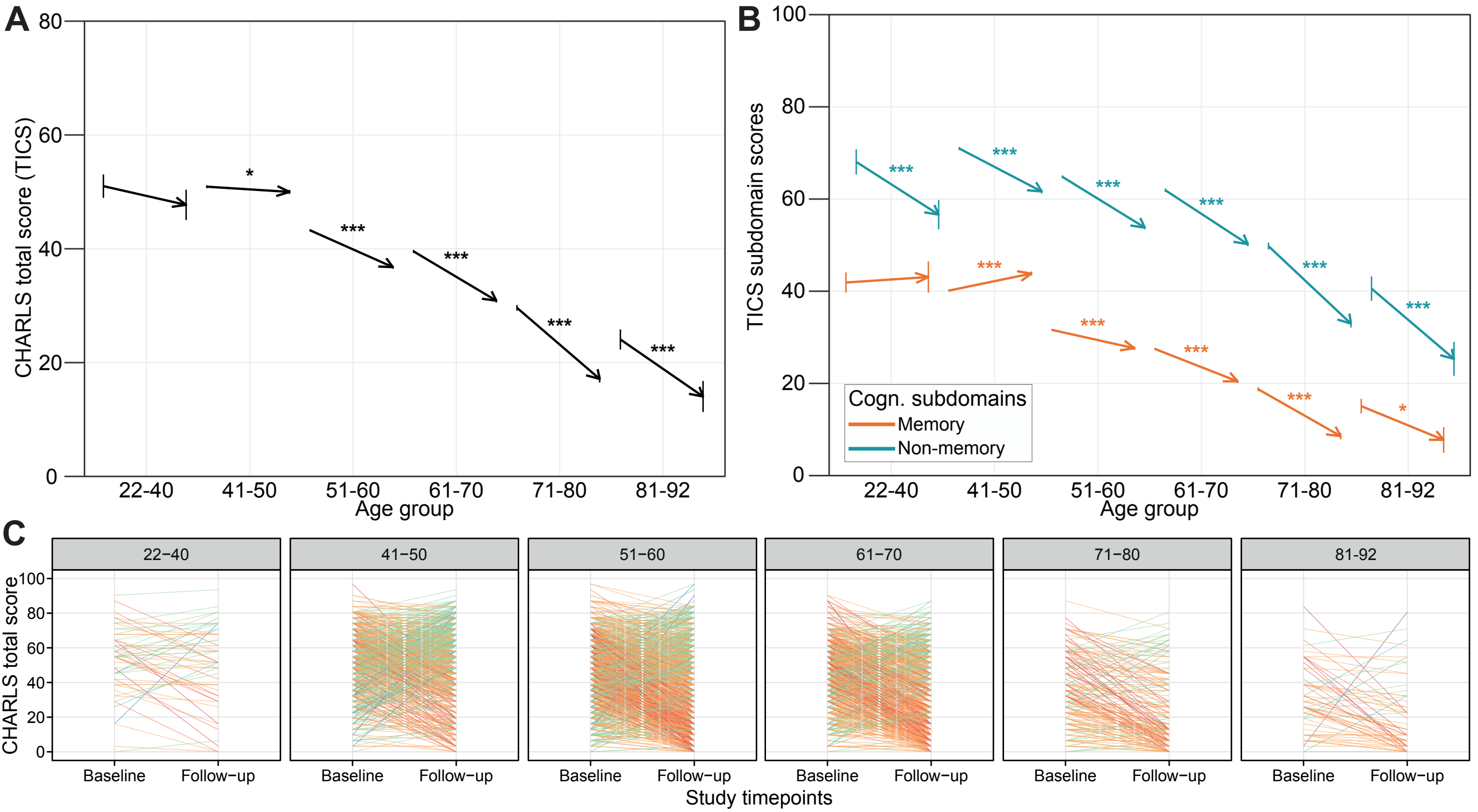
A
CHARLS total score (TICS)
Age group
22-40
41-50
51-60
61-70
71-80
81-92
*
***
B
TICS subdomain scores
Age group
Cogn. subdomains
Memory
Non-memory
C
CHARLS total score
Study timepoints
Baseline
Follow−up
22−40
41−50
51−60
61−70
71−80
81-92
