# Supplementary material for: Timing of cognitive decline onset and life-course trajectories A pooled longitudinal analysis of 14,389 adults across three cohorts
Source: J Prev Alzheimers Dis. 2026 Aug 7;13(9):100649. doi: 10.1016/j.tjpad.2026.100649 (PMC13475508; doi:10.1016/j.tjpad.2026.100649)
Supplement: Supplementary file 3 [file mmc5.pdf]

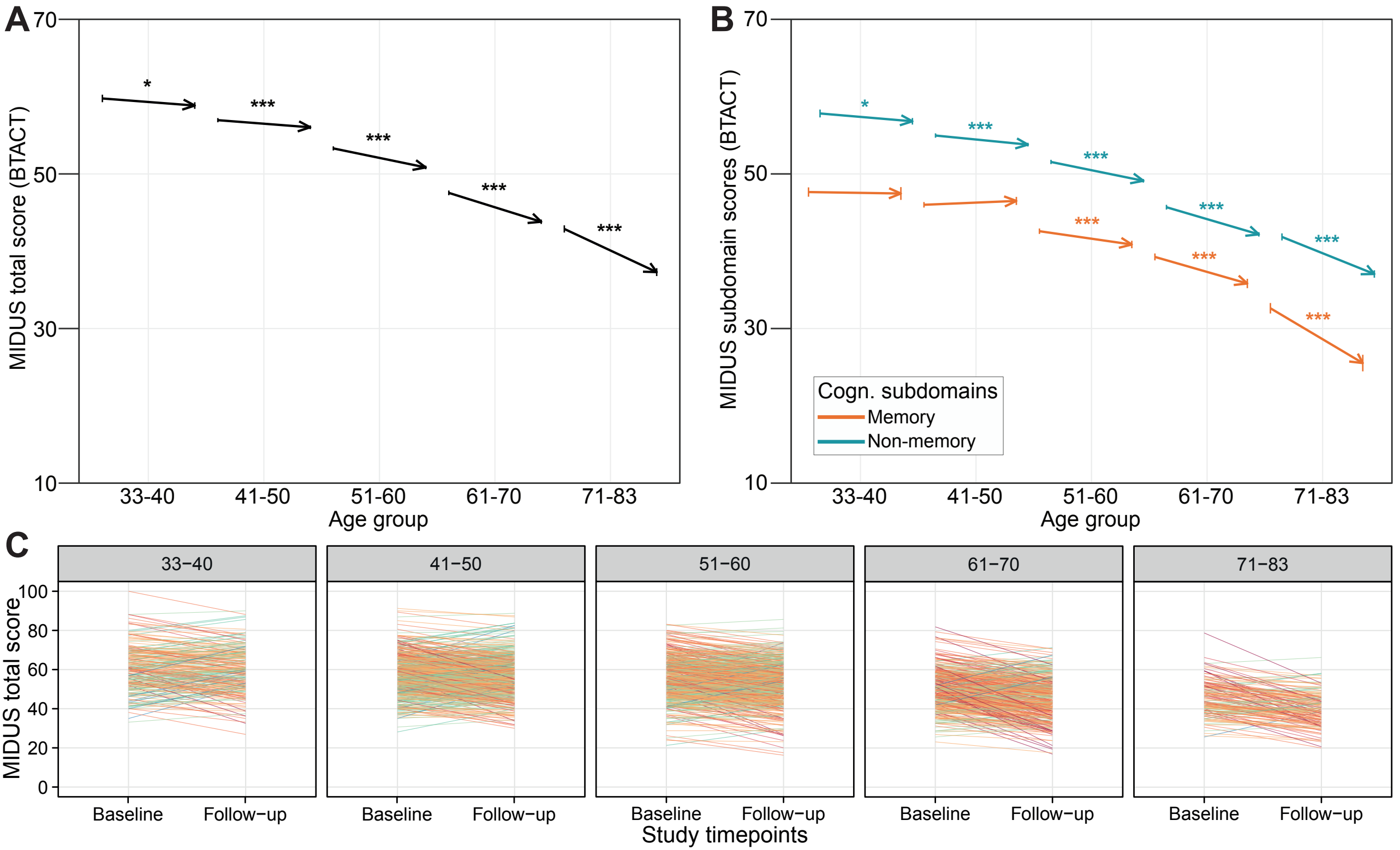

A
MIDUS total score (BTACT)
70
50
30
10
*
***
***
***
***
33-40
41-50
51-60
61-70
71-83
Age group
B
MIDUS subdomain scores (BTACT)
70
50
30
10
*
***
***
***
***
***
***
***
Cogn. subdomains
Memory
Non-memory
33-40
41-50
51-60
61-70
71-83
Age group
C
33−40
41−50
51−60
61−70
71−83
MIDUS total score
100
80
60
40
20
0
Baseline
Follow−up
Study timepoints
