# Supplementary material for: Timing of cognitive decline onset and life-course trajectories A pooled longitudinal analysis of 14,389 adults across three cohorts
Source: J Prev Alzheimers Dis. 2026 Aug 7;13(9):100649. doi: 10.1016/j.tjpad.2026.100649 (PMC13475508; doi:10.1016/j.tjpad.2026.100649)
Supplement: Supplementary file 4 [file mmc6.pdf]

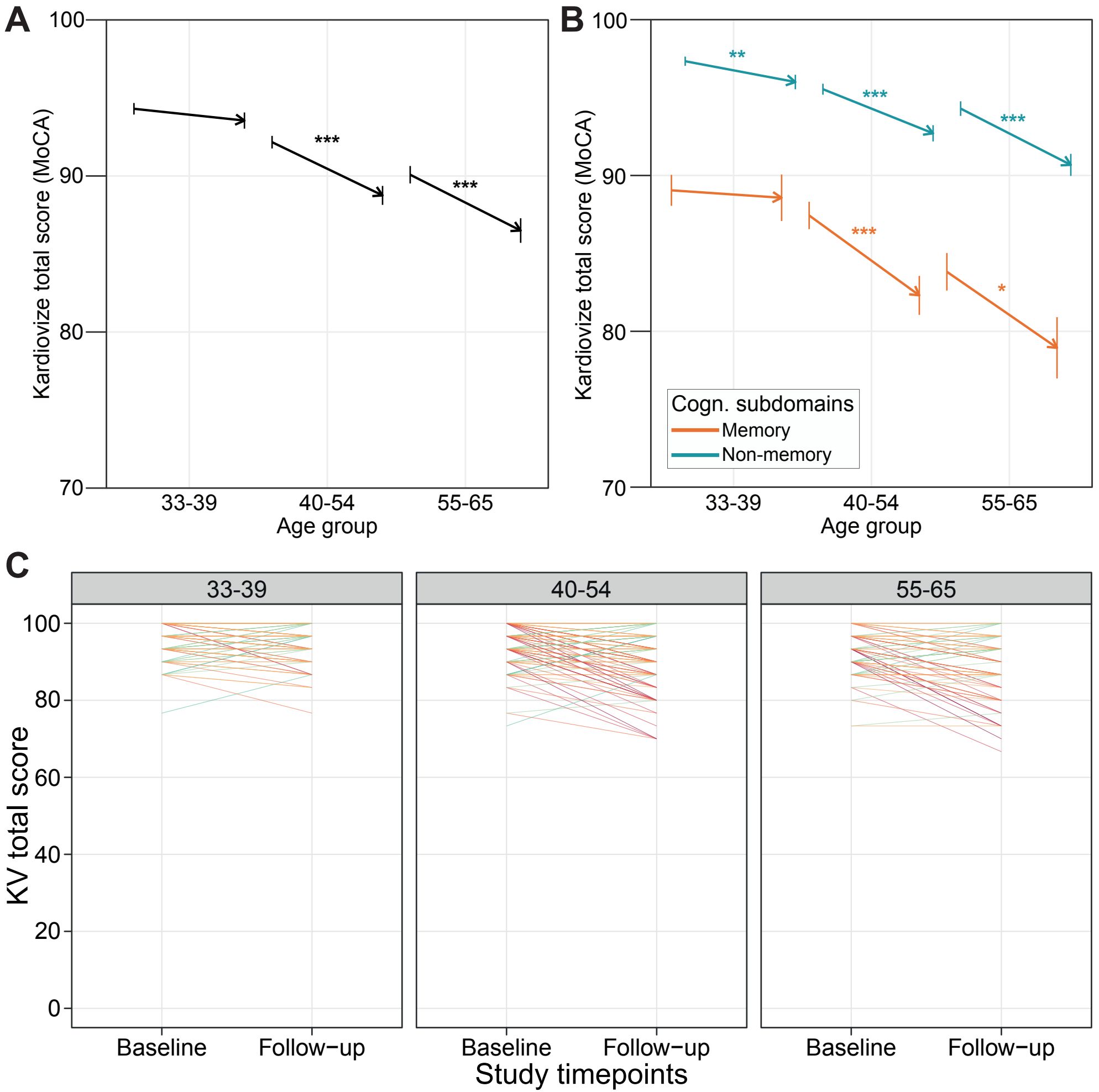
A
B
C
100
90
80
70
Kardiovize total score (MoCA)
***
**
*
33-39
40-54
55-65
Age group
Cogn. subdomains
Memory
Non-memory
KV total score
0
20
40
60
Baseline
Follow−up
Study timepoints
