# Supplementary material for: Timing of cognitive decline onset and life-course trajectories A pooled longitudinal analysis of 14,389 adults across three cohorts
Source: J Prev Alzheimers Dis. 2026 Aug 7;13(9):100649. doi: 10.1016/j.tjpad.2026.100649 (PMC13475508; doi:10.1016/j.tjpad.2026.100649)
Supplement: Supplementary file 5 [file mmc1.docx]

**Supplementary Figure 1. Comparison of cognitive domains assessed by the TICS-10, BTACT, and MoCA.**

The figure illustrates the overlap and differences in memory and non-memory domains across the three cognitive assessment instruments, which informed the harmonization strategy used in the present study.

**Supplementary Figure 2.** **Longitudinal cognitive change in the CHARLS cohort.**

(**A**) Estimated changes in harmonized total cognitive scores by baseline age group. Arrows indicate the direction and magnitude of change from baseline to follow-up, and error bars represent standard errors of the mean (SEM).

(**B**) Estimated longitudinal changes in aggregated memory and non-memory domains by baseline age group.

(**C**) Participant-level spaghetti plot showing individual trajectories of harmonized total cognitive scores from baseline to follow-up.

POMP, Percent of Maximum Possible harmonization method. Asterisks indicate statistically significant changes (*p ≤ 0.05, ***p ≤ 0.001).

**Supplementary Figure 3.** **Longitudinal cognitive change in the MIDUS cohort.**

(**A**) Estimated changes in harmonized total cognitive scores by baseline age group. Arrows indicate the direction and magnitude of change from baseline to follow-up, and error bars represent standard errors of the mean (SEM).

(**B**) Estimated longitudinal changes in aggregated memory and non-memory domains by baseline age group.

(**C**) Participant-level spaghetti plot showing individual trajectories of harmonized total cognitive scores from baseline to follow-up.

POMP, Percent of Maximum Possible harmonization method. Asterisks indicate statistically significant changes (*p ≤ 0.05, ***p ≤ 0.001).

**Supplementary Figure 4. Longitudinal cognitive change in the KV cohort.**

(**A**) Estimated changes in harmonized total cognitive scores by baseline age group. Arrows indicate the direction and magnitude of change from baseline to follow-up, and error bars represent standard errors of the mean (SEM).

(**B**) Estimated longitudinal changes in aggregated memory and non-memory domains by baseline age group.

(**C**) Participant-level spaghetti plot showing individual trajectories of harmonized total cognitive scores from baseline to follow-up.

POMP, Percent of Maximum Possible harmonization method. Asterisks indicate statistically significant changes (*p ≤ 0.05, **p ≤ 0.01, ***p ≤ 0.001).
